# Breath molecular feature profiling with machine learning for noninvasive diagnosis of gout: a single-center case-control study

**DOI:** 10.3389/fmolb.2026.1939939

**Published:** 2026-10-21

**Authors:** Zebin Cai, Xin Huang, Xuao Luo, Xiaoling Chen, Weikang Sun, Hua-nan Li

**Affiliations:** 1 School of Clinical Medicine, Jiangxi University of Chinese Medicine, Nanchang, Jiangxi, China; 2 Department of Orthopedics, Affiliated Hospital of Jiangxi University of Chinese Medicine, Nanchang, Jiangxi, China; 3 Scientific Research Department, Affiliated Hospital of Jiangxi University of Chinese Medicine, Nanchang, Jiangxi, China

**Keywords:** breath molecular features, extractive electrospray ionization mass spectrometry, gout, machine learning, metabolomics, noninvasive diagnosis

## Abstract

**Objective:**

Gout, a purine metabolism disorder with growing global prevalence, is currently diagnosed by ACR/EULAR criteria, yet complementary noninvasive tools remain limited. We evaluated whether breath molecular feature analysis could discriminate established gout patients from healthy controls.

**Methods:**

In this preliminary single-center study, breath samples were collected from 201 participants (99 established gout patients and 102 healthy controls). Breath molecular features were profiled by electrospray extraction ionization mass spectrometry (EESI-MS). Random forest machine-learning was applied to build classification models and identify discriminatory breath molecular features. Tongue-coating features and clinical characteristic were also compared between groups.

**Results:**

EESI-MS profiling of exhaled breath identified 12 differential breath molecular features as candidate gout biomarkers. The putative urate dimer was significantly upregulated, whereas the other 11 ions were downregulated. The BreathGOUT model based on breath molecular features achieved an AUC of 0.999 (95% CI 0.997–1.000) by 5-fold stratified cross-validation, with accuracy, sensitivity, and specificity all reaching 0.990. Unimodal decomposition analysis confirmed that BreathGOUT retained an AUC of 0.999, indicating that breath molecular features provided discriminatory information complementary to that captured by clinical characteristics alone. The trimodal integrated model, BreathGOUT-Plus, also yielded an AUC of 0.999; although it did not further improve diagnostic performance, it enhanced robustness against short-term confounding influences.

**Conclusion:**

In this case-control setting, machine-learning analysis of breath molecular features reliably discriminated established gout from healthy states. The model demonstrated excellent discriminative power and identified candidate biomarkers. Multimodal fusion improved clinical robustness and interpretability across Western and traditional Chinese medicine dimensions. This proof-of-concept supports future multicenter studies incorporating asymptomatic hyperuricemia, first-presentation arthritis, and other arthritides to assess broader clinical applicability.

## 1 Introduction

Gout is a highly prevalent metabolic rheumatic disease caused by dysregulated purine metabolism and impaired urate excretion, which together lead to hyperuricemia and subsequent monosodium urate crystal deposition that triggers a potent autoinflammatory response (FitzGerald, 2025). Clinically, it is characterized by recurrent acute arthritis, tophi, joint deformity, and renal impairment, and is frequently accompanied by metabolic syndrome and cardiovascular and cerebrovascular complications, thereby imposing a substantial burden on patient health (Keller and Mandell, 2022). Driven by changes in lifestyle, obesity, and population aging, the global burden of gout continues to rise. In 2020, an estimated 55.8 million individuals were affected worldwide, representing a 22.5% increase since 1990, and this number is projected to reach 95.8 million by 2050 (Kuo et al., 2015). The situation in China is similarly concerning: the prevalence of hyperuricemia among adults is 13.3%, the prevalence of gout is 1.1%, and more than 90 million individuals are affected, with a clear trend toward younger onset (Chinese Society of Endocrinology and Chinese Medical Association, 2020; Singh and Gaffo, 2020). Mounting evidence indicates that gout is not merely a consequence of hyperuricemia alone; rather, its pathogenesis involves multiple interrelated processes, including disturbed urate handling and excessive activation of inflammatory pathways, and is closely linked to metabolic syndrome (FitzGerald, 2025; Zhu et al., 2012). Therefore, elucidating the pathogenic mechanisms of gout and developing safe and effective interventions are of considerable importance for improving clinical management and alleviating the disease burden.

At present, gout diagnosis and management primarily rely on the ACR/EULAR criteria, supported by serum urate testing, imaging, and crystal identification in synovial fluid, which together provide high specificity and established clinical authority (Dalbeth et al., 2021). However, this diagnostic framework remains far from ideal. Serum urate levels are easily confounded and may yield false-negative results, particularly in patients with early metabolic abnormalities, whereas invasive procedures are unsuitable for large-scale screening or longitudinal follow-up. In this context, noninvasive metabolomic assessment of VOCs in urine and breath offers a compelling alternative, as it can sensitively reflect purine metabolic disruption and inflammatory stress, both of which lie at the core of gout pathobiology. Indeed, experimental studies have shown that VOCs may participate in gout progression by promoting inflammation and suppressing renal tubular urate excretion (Hu et al., 2024). This approach is noninvasive, repeatable, and potentially suitable for early warning and quantitative monitoring, thereby addressing several critical limitations of conventional diagnostics (Lu et al., 2025). Nevertheless, VOC biomarkers remain insufficiently disease-specific, and no standardized analytical workflow or diagnostic threshold has yet been established; as a result, VOC-based testing can currently serve only as an adjunctive screening tool rather than a replacement for the gold-standard diagnostic criteria (Cen et al., 2024). Thus, while conventional methods remain essential for definitive diagnosis, VOC analysis represents an important complementary strategy, and their integration may provide a more precise framework for full-course gout management.

Breath VOCs profiling has been increasingly applied to inflammatory diseases. In inflammatory bowel disease, a seven-breath VOCs panel detected by GC–MS discriminated patients from non-IBD controls with AUCs of 1.000, 0.978, and 0.966 in successive validation stages, and several markers correlated with disease activity (Zhang et al., 2025). Breathomics studies in asthma have similarly established that exhaled breath VOCs are sensitive to underlying airway inflammation and can differentiate patients from healthy controls while supporting phenotypic stratification (Azim et al., 2019). In hepatobiliary disease, a panel of 20 breath VOCs distinguished primary sclerosing cholangitis from IBD controls with an AUC of 0.84, and detected early-stage cases even when alkaline phosphatase was normal (van Vorstenbosch et al., 2024). Together, these findings indicate that chronic inflammation—whether mucosal, airway, or biliary—produces characteristic volatile metabolite patterns accessible via noninvasive breath sampling. It is therefore mechanistically plausible that this paradigm extends to gout, a disorder driven by monosodium urate crystal deposition and purine metabolic dysregulation. Mechanistic studies linking breath VOCs production to metabolic perturbations and oxidative stress during inflammation (Chou et al., 2024; Wei et al., 2026) support the view that breath VOCs have the potential to reflect purine metabolic dysregulation and inflammatory activity, making them a promising target for noninvasive gout diagnosis. Notably, these studies were performed by GC–MS, which is highly sensitive and specific for volatile compounds. Ambient ionization techniques such as EESI-MS provide complementary coverage, extending breath analysis to semivolatile and non-volatile metabolites and thereby offering a broader molecular window into the same pathophysiological processes.

Building on this broader concept, we evaluate the diagnostic potential of breath molecular features in gout, this study addressed both the choice of analytical platform and the value of multimodal data integration. EESI-MS, a newly developed ambient ionization technique (Qin et al., 2023), enables rapid and noninvasive detection of trace molecular features without sample pretreatment, and offers strong anti-interference capability for the identification of gout-related metabolic signatures (Hofstadler and Sannes-Lowery, 2006). Compared with conventional platforms such as Gas Chromatography-Mass Spectrometry (GC-MS)and Liquid Chromatography-Mass Spectrometry (LC-MS), EESI-MS supports *in situ*, real-time breath analysis and covers a broader range of candidate biomarkers, making it particularly suitable for large-scale screening and dynamic monitoring; however, its performance in precise qualitative and quantitative characterization still requires improvement (Ke et al., 2019; Li et al., 2009). Beyond the selection of an analytical platform, the clinical complexity of gout itself motivates a multidimensional diagnostic approach. Gout-related pathophysiology spans metabolic dysregulation, epidemiological-clinical characteristics, and traditional Chinese medicine (TCM) syndrome patterns. Breath molecular features profiling captures the metabolic dimension; clinical characteristics capture the epidemiological dimension; and tongue-coating characteristics capture the TCM syndrome dimension, which is closely associated with gout onset and subtype classification (Li R. et al., 2023). Therefore, in this study, we developed both a mass spectrometry–only classification model and an integrated multimodal model incorporating clinical and tongue features, and we systematically compared their discriminative performance in a proof-of-concept case-control setting. The aim was to assess whether multi-source data integration could enhance the robustness of breath molecular features profiling for distinguishing established gout patients from healthy controls, as a foundation for future clinical validation.

Primary hypothesis and performance measures. We hypothesized that machine-learning analysis of exhaled breath molecular features would discriminate established gout patients from healthy controls in this case-control sample. The prespecified primary performance measure was the area under the receiver-operating characteristic curve (AUC) of the BreathGOUT model, assessed by 5-fold stratified cross-validation with pooled out-of-fold predictions. Secondary performance measures included AUPRC, accuracy, sensitivity, and specificity; the trimodal BreathGOUT-Plus model was evaluated as a secondary analysis to assess the incremental value of multi-source data integration.

## 2 Materials and methods

### 2.1 Study design

The cohort was recruited at the Affiliated Hospital of Jiangxi University of Chinese Medicine between December 2025 and March 2026. In this non-randomized study, consecutive eligible volunteers were enrolled to minimize selection bias. Healthy controls were recruited from routine health examination programs and were required to have normal joint mobility and sensation, normal serum urate levels, and no history of chronic renal or metabolic disease.

A total of 201 participants were included, comprising 102 healthy controls and 99 gout patients. No participants were excluded due to missing breath spectra, poor tongue-image quality, or incomplete clinical variables. Model performance was evaluated by 5-fold stratified cross-validation, with feature selection conducted independently within each training fold to prevent data leakage. All models were trained and tested on the identical cohort of 201 participants.(Figure 1)

**FIGURE 1:**
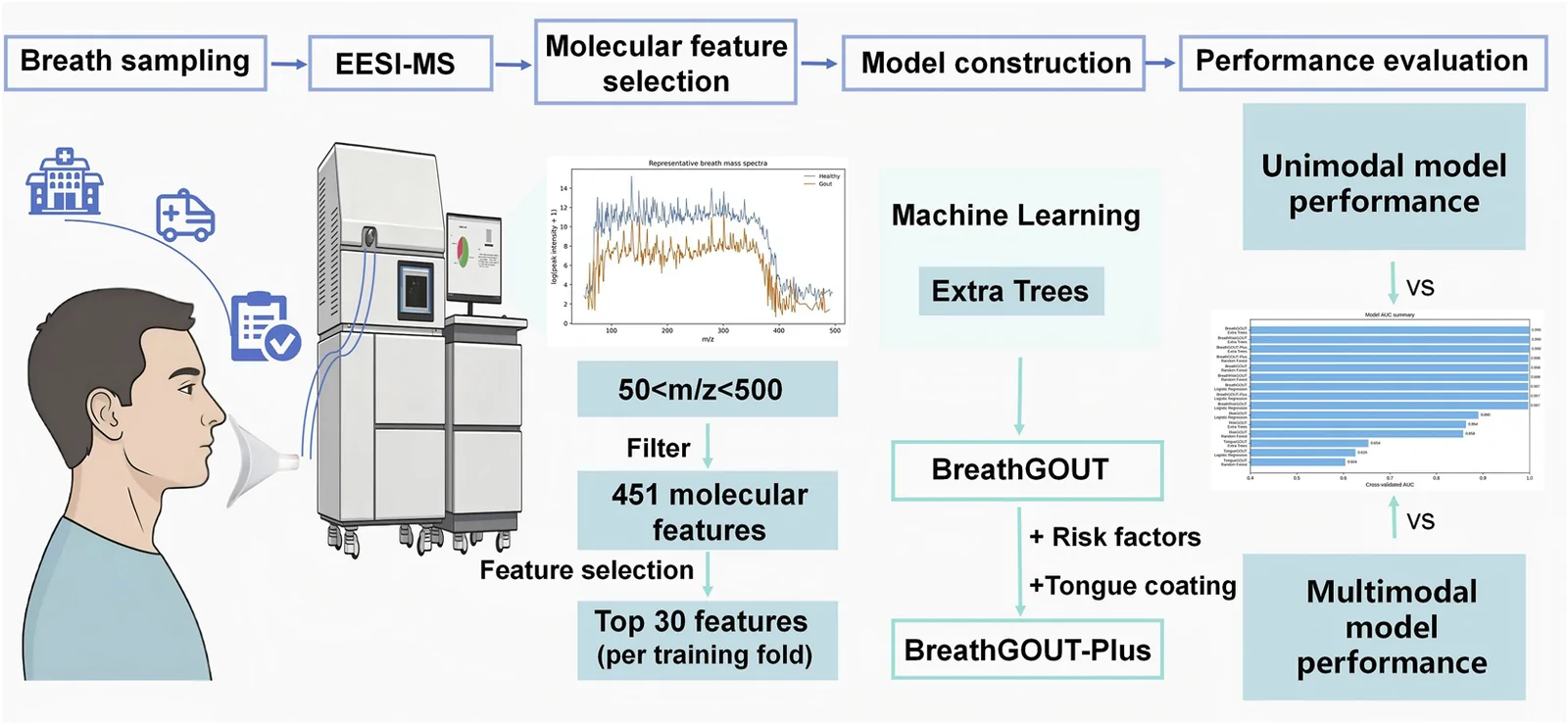
Data analysis workflow of the BreathGOUT and BreathGOUT-Plus models.

#### 2.1.1 Inclusion criteria for healthy controls

1. Male individuals with normal findings on routine physical examination or no clearly diagnosed disease;
2. Age between 18 and 80 years;
3. No history of major cardiac, hepatic, renal, neurological, or infectious disease;
4. Willingness to participate and provide written informed consent (see Supplementary Materials).

#### 2.1.2 Inclusion criteria for patients with gout

1. Male patients with a confirmed diagnosis of gout, classified according to the 2015 ACR/EULAR gout classification criteria (Neogi et al., 2015). All patients fulfilled the entry criterion of at least one episode of pain, swelling, or tenderness in a peripheral joint or bursa. Patients were classified as having gout if monosodium urate (MSU) crystals were detected by polarized light microscopy in synovial fluid aspirated from a symptomatic joint or bursa, or in a tophus (sufficient criterion). Participants who did not meet the sufficient criterion were scored across the clinical (pattern of joint involvement, 0–2 points; characteristics of symptomatic episodes, 0–3; time course, 0–2; clinical tophus, 0–4), laboratory (serum urate level, −4 to +4; synovial fluid analysis during an episode, −2 to 0), and imaging (ultrasound double-contour sign, dual-energy computed tomography urate deposition, and radiographic gout-related erosion, each 0–4) domains. A total score of ≥8 (maximum 23) was required for classification as gout.

2.Age between 18 and 80 years;

3.Willingness to undergo breath testing and provide written informed consent (see Supplementary Materials).

### 2.2 Breath sample collection and analysis

Standardized sampling procedures were established to minimize confounding effects.Participants were instructed to avoid smoking, alcohol consumption, and food intake for at least 1 h before sampling. Immediately before collection, participants rinsed their mouths thoroughly with purified water to reduce the influence of residual diet, smoking, and alcohol. All samples were collected at the same site to minimize environmental variability. After a deep nasal inspiration, participants exhaled steadily and forcefully into the EESI-MS device for 20 s, after which the corresponding mass spectrum was acquired.

Breath molecular feature profiling was performed on an LTQ-Velos linear ion trap mass spectrometer (Thermo Fisher Scientific, United States) coupled with an EESI ionization source (model HY20221011MS, Henyao Technology, China). Before each analysis session, the instrument was evacuated for at least 8 h. The ambient temperature was maintained at 18 °C–25 °C (preferably 21 °C), and the relative humidity was kept at 30%–60% RH without condensation. The extraction solvent consisted of 47.5% methanol, 47.5% water, and 5% acetic acid, delivered at a flow rate of 4.1 μL/min. The voltage was set to 3.0 kV, and high-purity helium and nitrogen were used at a gas pressure of 0.6 MPa. All spectra were acquired in positive-ion mode (ITMS + c ESI Full ms [50.00–950.00]) with Normal mass resolution. Prior to each batch, external mass calibration was performed following the manufacturer-recommended tuning protocol; the mass tolerance for feature matching was set to ±0.5 Da.

Quality control and reproducibility measures included solvent blanks run at regular intervals to monitor background stability and assess carry-over. No pooled QC samples or isotopically labeled internal standards were used, as absolute quantification was not the objective of this untargeted profiling study. Technical replicates were not performed owing to the preliminary nature of this study. All samples were analyzed within a single batch; therefore, batch correction was unnecessary.

Because EESI-MS ionizes analytes directly from the exhaled breath stream, including both gas-phase molecules and aerosol-bound or droplet-phase species (Ke et al., 2019; Li et al., 2009), the term “breath molecular features” is used throughout to refer to the detected m/z ion signals. Raw mass spectra were preprocessed using an in-house Python algorithm. Features were extracted across the m/z range 50–500 using a 1-Da grid with a ±0.5-Da window to approximate peak intensity/area, yielding 451 breath molecular features. Background subtraction was applied to remove persistent signals arising from solvent and ion-source interference. Peak areas were normalized by total ion current (TIC) and subsequently log1p-transformed to stabilize variance. Ion features with missing values in more than 20% of samples were excluded; remaining missing values were imputed with half the minimum observed value. Given the limited structural identification capability of EESI-MS, candidate ions were inferred on the basis of m/z values, peak areas, and the physicochemical properties of potential biomarkers. Breath metabolic components were searched by matching m/z values against a human breath metabolomics database, and candidate assignments were confirmed based on detectable concentration, physicochemical properties, and previously reported gout-related breath VOCs biomarkers.

### 2.3 Tongue-feature acquisition and analysis

In accordance with authoritative domestic and international standards for digital tongue diagnosis, a standardized smartphone-based tongue imaging system was independently established for unified image acquisition across all participants, thereby ensuring data consistency and reproducibility. After breath sample collection, participants were instructed to sit upright, relax, and protrude the tongue naturally under the guidance of the investigators.

Images were acquired using a high-definition smartphone camera that met industry standards under illumination conditions of 5000 K color temperature and 4,800 lx luminance. The core camera settings were standardized as follows: beautification, filters, HDR, and color correction were disabled; aperture was fixed at f/1.8; ISO was set to 100; and shutter speed was set to 1/100 s. Each participant was photographed three times, and images were saved in JPG format. Strict image quality control was performed, and blurred, overexposed, reflective, tongue-coating-stained, morphologically abnormal, or obstructed/distorted images were excluded. Only qualified images were retained for subsequent analysis. This study was approved by the Ethics Committee, and all participants provided written informed consent in accordance with ethical standards for clinical research.

### 2.4 Statistical and bioinformatics analysis

Descriptive statistics were calculated for all baseline characteristics. Continuous variables are presented as mean ± standard deviation (SD) according to distribution and were assessed using the Shapiro–Wilk test. Categorical variables are reported as counts and percentages. To compare individual variables between the gout and control groups, independent-samples t-tests were applied to normally distributed data, whereas Mann–Whitney U tests were used for non-normally distributed data. All tests were two-sided, and P < 0.05 was considered statistically significant. To identify gout-associated factors, Candidate biomarker screening was performed on the complete dataset by combining pairwise spectral comparison and correlation analysis with volcano-plot visualization. Differential breath molecular features were identified by nonparametric testing with false discovery rate (FDR) correction. These differential features served as candidate biomarkers for biological interpretation only and were not used as the input feature set for model construction.

Three machine-learning models were developed: logistic regression, random forest, and Extra Trees. For each data configuration, model performance was evaluated by 5-fold stratified cross-validation. In each fold, feature selection and preprocessing were performed on the training subset and applied to the validation subset to prevent data leakage. Predicted probabilities across all five validation folds were aggregated to compute the final AUC and other diagnostic metrics.

For high-dimensional breath mass-spectrometry features, all breath molecular features were retained at the initial modeling stage. Then, within each training fold of cross-validation, the top 30 features were selected based on univariate statistical ranking and were entered into the classification model to reduce overfitting in the setting of small-sample, high-dimensional data. Accordingly, the model input comprised the full set of 451 breath molecular features, and feature selection was restricted to the training subset of each fold, ensuring that no information from the validation folds entered the feature-selection process. Statistical analyses were performed using SPSS 25 and R 4.3.3, and machine-learning tasks were implemented in Python 3.11 within VS Code 1.123.0. Unless otherwise specified, default parameters were used.

## 3 Results

### 3.1 Baseline clinical characteristic

The study cohort comprised 201 participants, including 99 patients with gout and 102 healthy controls. Age was comparable between the two groups, although the difference did not reach the conventional threshold for statistical significance, whereas Body Mass Index (BMI) was significantly higher in the gout group (P < 0.001). Serum urate levels were significantly higher in gout patients than in healthy controls (P < 0.001). As shown in Table 1, the gout group had a higher prevalence of alcohol consumption (P < 0.001), and the difference in smoking history was also statistically significant (P = 0.012). The difference in family history of gout approached statistical significance.

**TABLE 1:** Analysis of clinical characteristic in subjects.

Variables	Healthy group	Gout group	P Value
Age1, years	49.00 ± 11.78	45.20 ± 14.35	0.059
BMI1, kg/m2	22.07 ± 3.44	27.88 ± 4.64	<0.001
Serum urate1, μmol/L	353.57 ± 55.38	514.81 ± 86.13	<0.001
Smoking History2	27 (26.5%)	44 (44.4%)	0.012
History of alcohol consumption2	33 (32.4%)	65 (65.7%)	<0.001
Family history of gout2	12 (11.8%)	23 (23.2%)	0.050

^1^Continuous variables are presented as mean ± SD;^2^Categorical variables as n (%)

### 3.2 Differences in breath molecular features and candidate biomarker screening

Breath samples from all 201 participants were analyzed by EESI-MS to generate breath mass spectra. To visualize and preserve the authenticity of global spectral morphology, raw spectra were binned according to mass-to-charge ratio (m/z) to generate median-intensity curves (Figure 2). Visual inspection of the resulting mass spectra revealed marked differences in breath molecular features between the gout and healthy groups. A total of 451 breath molecular features were quantified and constituted the full candidate feature pool for modeling. Differential ions were further screened using nonparametric testing combined with FDR correction and volcano-plot analysis (Figure 3), and pairwise correlations among the selected breath molecular features were visualized to assess potential redundancy. (Figure 4). Ultimately, 12 key m/z features were selected as candidate biomarkers.

**FIGURE 2:**
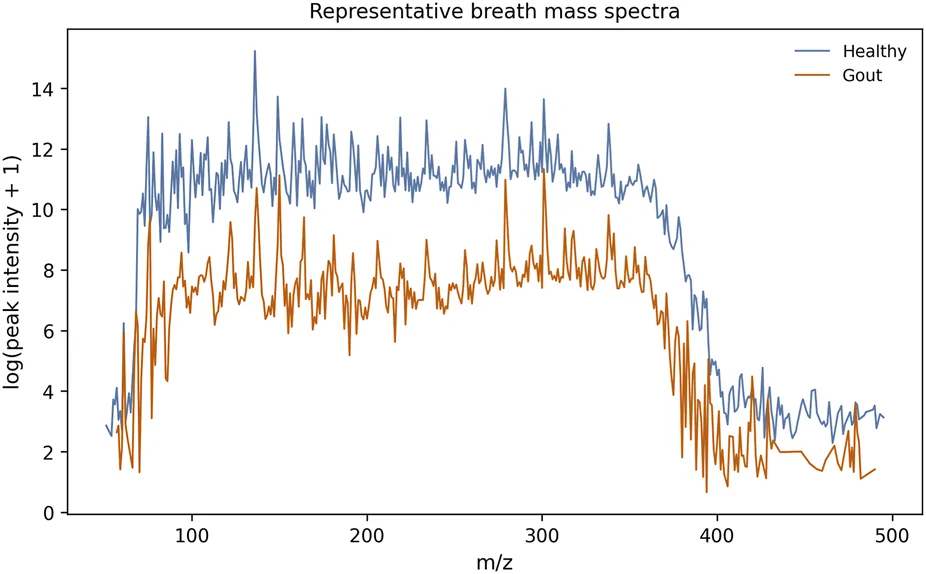
Distinct breath molecular features characteristics between the gout and healthy groups. Blue indicates healthy participants, and orange indicates patients with gout.

**FIGURE 3:**
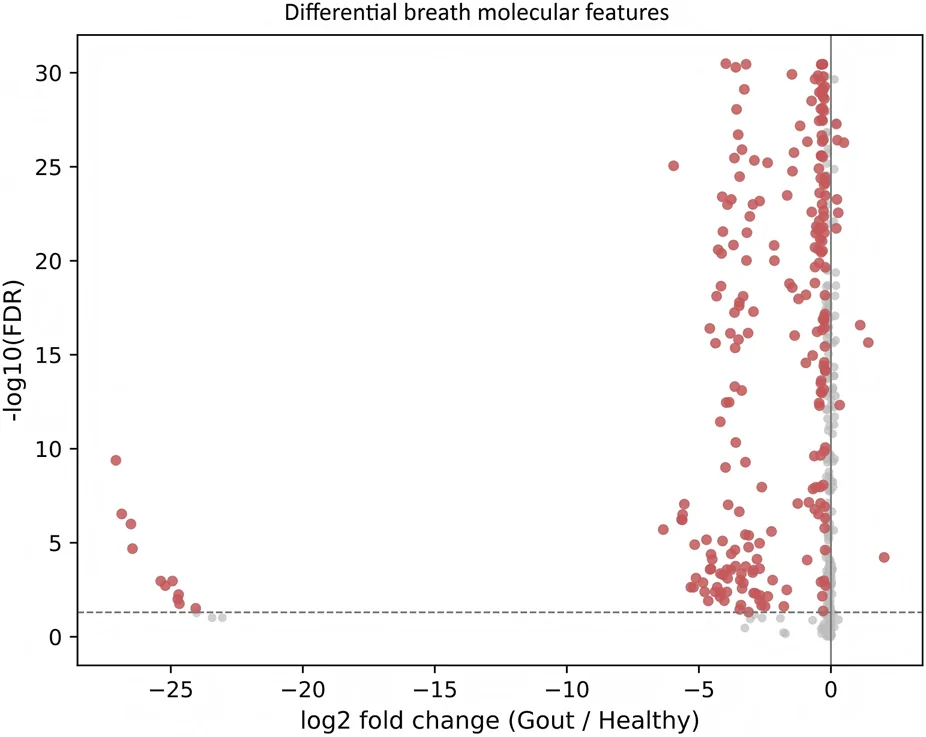
Volcano plot of differential breath molecular features. Red dots indicate ion peaks with greater statistical significance.

**FIGURE 4:**
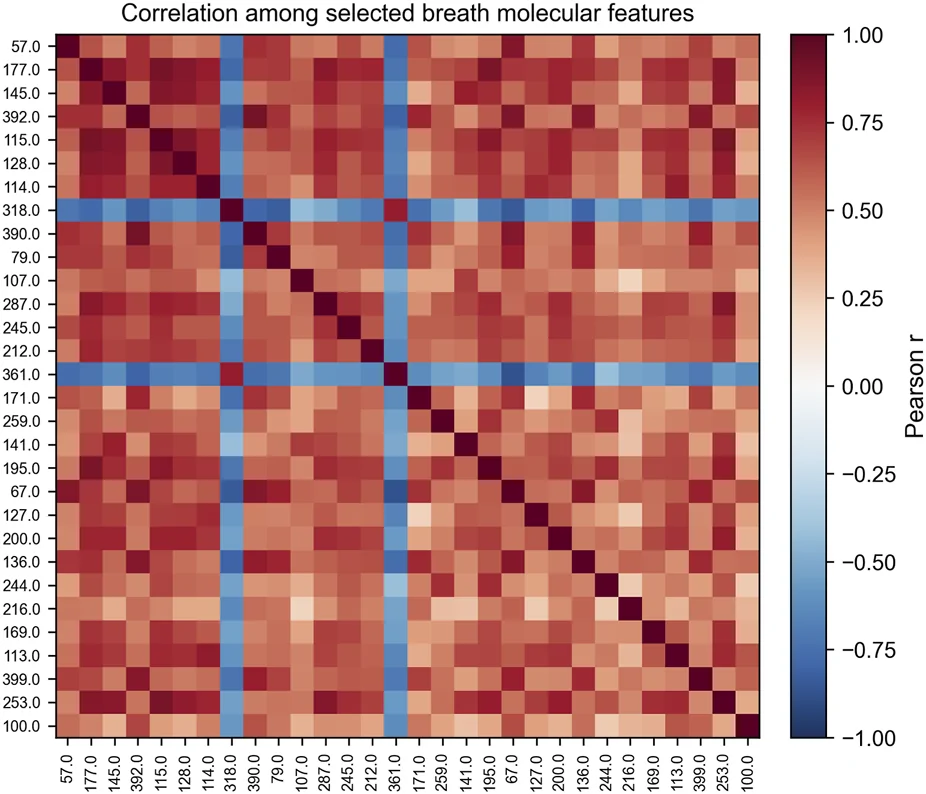
Pearson correlation heatmap of selected breath molecular features m/z values in exhaled breath samples. The color gradient represents the Pearson correlation coefficient ®: deep red indicates a strong positive correlation (r = 1), and deep blue indicates a strong negative correlation (r = −1). Most volatile organic compound ion features showed linear positive correlations, whereas the ions with m/z values of 318.0, 361.0, and 100.0 were significantly negatively correlated with most other VOCs features and exhibited strong mutual negative correlations.

We further examined the top 12 breath molecular features with distinct m/z values, which discriminated patients with gout from healthy individuals. Based on m/z, peak area, correlation with other ions, and the human breath metabolomics database (Kuo et al., 2020),these breath molecular features were tentatively assigned to alkane derivatives (m/z 57.0), aromatic hydrocarbon derivatives (m/z 79.0), cresol (m/z 107.0), hexanoic acid (m/z 114.0), levulinic acid (m/z 115.0), cycloalkanol (m/z 128.0), inosine (m/z 145.0), hippuric acid (m/z 177.0), quercetin (m/z 287.0), urate dimer (m/z 318.0), triglyceride (m/z 390.0), and chenodeoxycholic acid (m/z 392.0) (Table 2). Of note, these putatively annotated molecules include both volatile species and non-volatile or semivolatile metabolites, which is consistent with the ability of EESI-MS to sample aerosol-phase and droplet-phase constituents in addition to true VOCs.

**TABLE 2:** Top 12 differential breath molecular features between gout patients and healthy subjects.

m/z	Tendency in gout	Potential breath metabolites	mean_healthy	mean_gout	log2FC	FDR	AUC	p_value
57.000	↓	alkane derivative	1.066	0.068	−3.980	3.29e-31	0.972	<0.001
177.000	↓	hippuric acid	9.150	7.376	−0.311	3.53e-31	0.992	<0.001
145.000	↓	inosine	7.552	6.118	−0.304	3.53e-31	0.991	<0.001
392.000	↓	chenodeoxycholic acid	3.647	0.393	−3.213	3.53e-31	0.978	<0.001
115.000	↓	levulinic acid	7.538	5.849	−0.366	3.64e-31	0.989	<0.001
128.000	↓	cycloalkanol	7.824	6.084	−0.363	3.64e-31	0.989	<0.001
114.000	↓	hexanoic acid	7.424	5.376	−0.466	3.64e-31	0.988	<0.001
318.000	↑	urate dimer	7.332	9.307	0.344	5.15e-31	0.987	<0.001
390.000	↓	triglyceride	2.273	0.187	−3.604	5.15e-31	0.964	<0.001
79.000	↓	aromatic hydrocarbon derivative	7.083	2.545	−1.477	1.22e-30	0.978	<0.001
107.000	↓	cresol	8.343	5.938	−0.491	1.41e-30	0.982	<0.001
287.000	↓	quercetin	8.442	6.906	−0.290	1.58e-30	0.982	<0.001

We also evaluated the discriminative performance of these 12 breath molecular features individually and in combination, as shown in Figure 5. To identify the most influential biomarkers corresponding to the top 12 differential breath molecular features, features were ranked by importance in the best-performing Extra Trees model. Signals at m/z 57.0, 79.0, 107.0, 114.0, 115.0, 128.0, 145.0, 177.0, 287.0, 390.0, and 392.0 were downregulated, whereas the signal at m/z 318.0 was upregulated. Figure 6 visually illustrates the distinct expression patterns of these top compounds. The FDR values for the top 12 specific breath molecular features were extremely low, and the AUC of most single features exceeded 0.90, indicating a strong discriminative signal in breath mass spectra.

**FIGURE 5:**
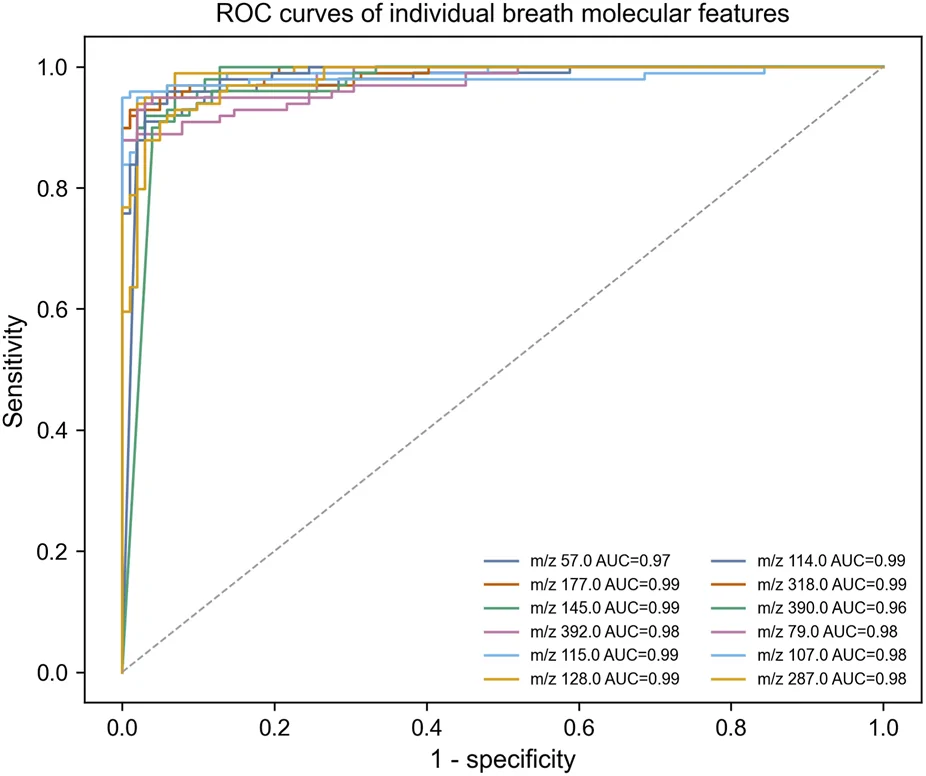
Single-feature ROC curves for the top 12 breath molecular features. Several individual ion peaks demonstrated strong diagnostic discriminatory performance.

**FIGURE 6:**
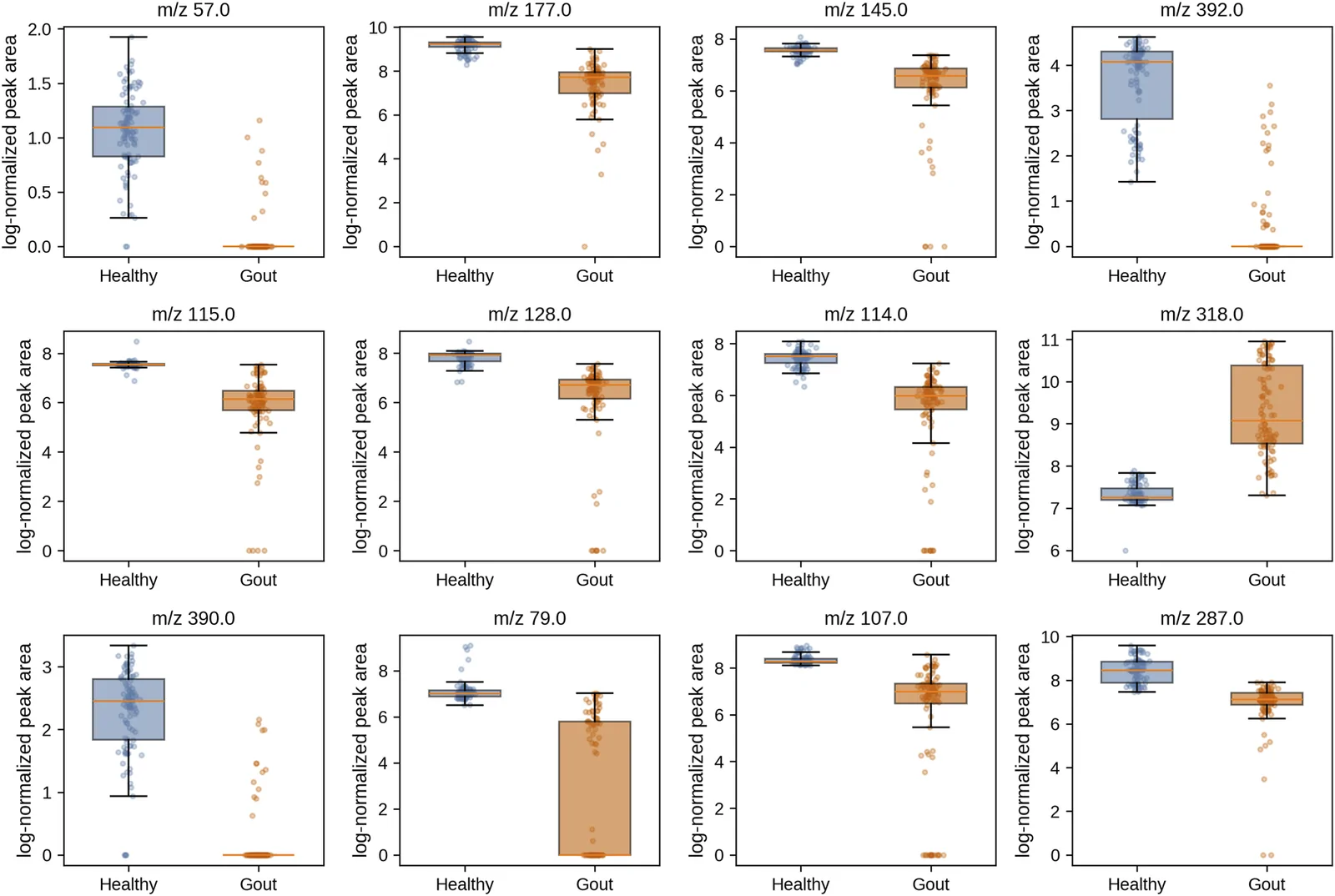
Boxplots of the top 12 breath molecular features in the healthy and gout groups. Most candidate peaks showed clear between-group separation.

### 3.3 Tongue and sublingual image feature analysis

Two standardized-view tongue images were acquired for each subject: the tongue-body view and the sublingual view. The images were converted to the RGB color space, resized to 128 × 128 pixels, and cropped by 8% to remove border regions. A brightness- and redness-based mask was constructed to segment valid tongue-coating pixels. The proportion of retained pixels was recorded as tongue_mask_ratio, a quality-control metric that reflects the coverage of the region-of-interest (ROI). Fourteen features were extracted from the masked region for each view: means and standard deviations of the R, G, and B channels (6 features), four color indices (redness index, yellow-coating index, whiteness index, and darkness index), mean HSV saturation, ROI coverage ratio, and two grayscale texture metrics (grayscale standard deviation and the Q90-Q10 percentile difference). In total, 28 tongue-image features were obtained per subject, all of which were fed into the TongueGOUT model (Supplementary Table 2). Among these, 12 clinically interpretable features were presented in Figure 7 and Table 3.

**FIGURE 7:**
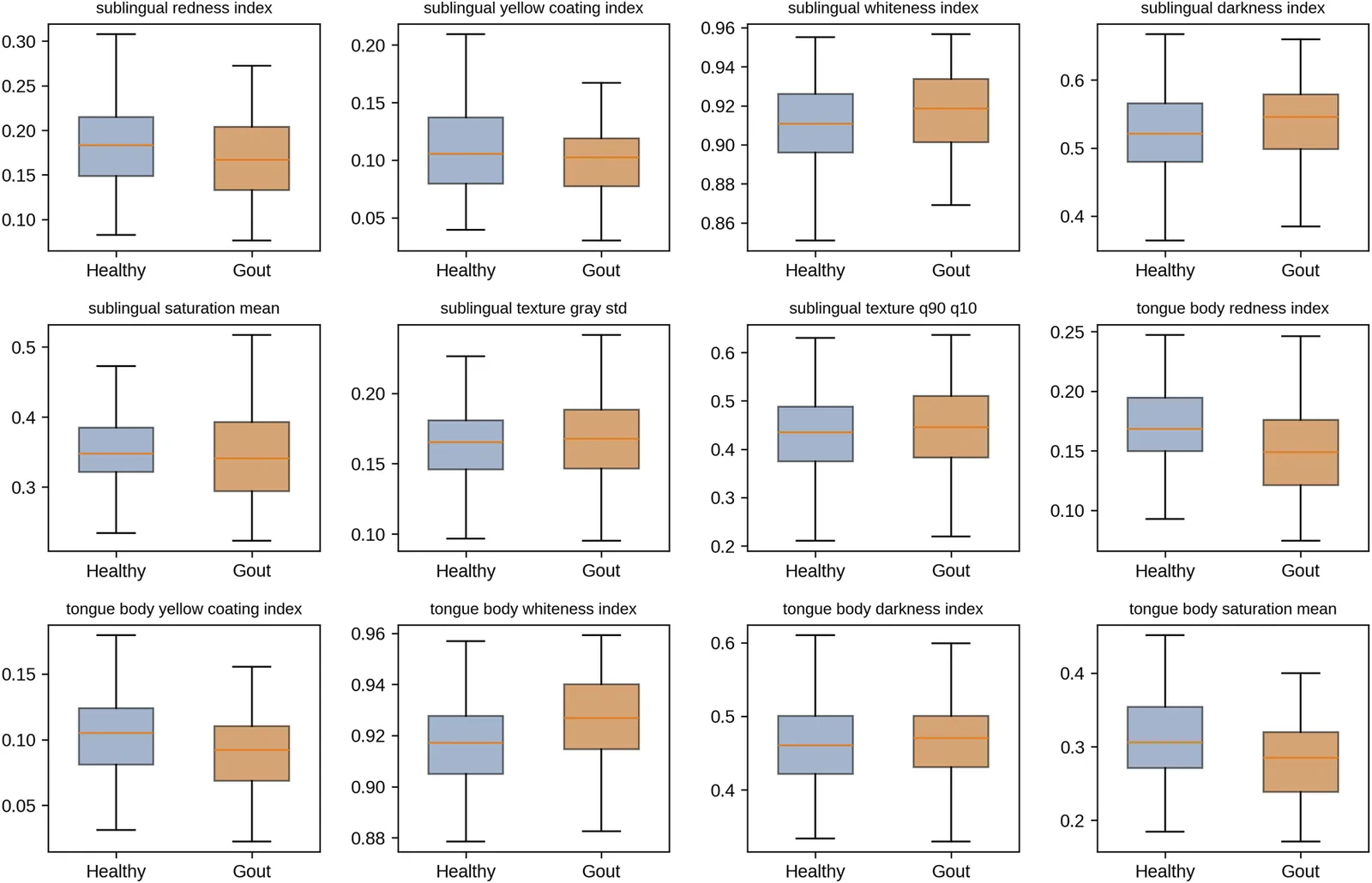
Quantitative differences in tongue image features. This figure shows the inter group differences between gout patients and healthy controls in 12 characteristics, including color, tongue coating, and texture.

**TABLE 3:** Comparison of 12 tongue features between gout patients and healthy control group.

Feature	Healthy (n = 102)	Gout (n = 99)	P Value	FDR q value	Effect size (r)
Sublingual Redness Index	0.183 (0.149–0.215)	0.167 (0.133–0.204)	0.0445	0.0763	−0.142
Sublingual Yellow Coating Index	0.105 (0.080–0.137)	0.103 (0.077–0.119)	0.1081	0.1621	−0.113
Sublingual Whiteness Index	0.911 (0.896–0.926)	0.919 (0.901–0.934)	0.0367	0.0742	0.147
Sublingual Darkness Index	0.521 (0.480–0.566)	0.546 (0.498–0.579)	0.0371	0.0742	0.147
Sublingual Saturation Mean	0.348 (0.322–0.385)	0.341 (0.294–0.393)	0.2579	0.3094	−0.08
Sublingual Texture Gray Std	0.165 (0.146–0.181)	0.168 (0.146–0.188)	0.447	0.447	0.054
Sublingual Texture Q90 Q10	0.435 (0.375–0.488)	0.446 (0.383–0.510)	0.2543	0.3094	0.08
Tongue Body Redness Index ***	0.168 (0.150–0.195)	0.149 (0.121–0.176)	<0.001	<0.001	−0.266
Tongue Body Yellow Coating Index *	0.105 (0.081–0.124)	0.092 (0.069–0.110)	0.0105	0.0314	−0.181
Tongue Body Whiteness Index ***	0.917 (0.905–0.928)	0.927 (0.915–0.940)	<0.001	<0.001	0.275
Tongue Body Darkness Index	0.461 (0.421–0.501)	0.470 (0.431–0.501)	0.3301	0.3602	0.069
Tongue Body Saturation Mean **	0.306 (0.271–0.354)	0.285 (0.238–0.320)	0.0011	0.0043	−0.231

Data are presented as median (interquartile range). Between-group comparisons were performed using the Mann–Whitney U test, and multiple comparisons were corrected by the Benjamini–Hochberg false discovery rate (FDR) procedure. The effect size was reported as the ran-biserial correlation. Significance markers: *q < 0.05, **q < 0.01, ***q < 0.001.

Group comparisons were performed using the Mann-Whitney U test with Benjamini-Hochberg false-discovery-rate (FDR) correction. Three tongue-body features showed significant between-group differences after FDR correction (Table 4). The tongue-body redness index was significantly lower in gout patients (FDR q < 0.001, r = −0.266), consistent with the dull-red tongue appearance commonly observed in patients with gout. The tongue-body whiteness index was significantly elevated (FDR q < 0.001, r = +0.275), suggesting increased accumulation of white tongue coating in gout patients. Tongue-body saturation was also decreased (FDR q = 0.004, r = −0.231), indicating reduced chromatic vividness of the tongue body. Notably, none of the seven sublingual-view features reached statistical significance following FDR correction, indicating that the tongue body provides stronger discriminative information than the sublingual region in this cohort. No between-group difference was found in tongue_mask_ratio (P > 0.05 for both views), confirming comparable ROI coverage between groups and eliminating bias arising from segmentation quality. These findings suggest that tongue-image features can serve as a complementary analytical modality within the multimodal BreathGOUT-Plus framework.

**TABLE 4:** Best model performance for each data set.

Dataset	Top model	Features	Thr_Youden	AUC-ROC (95% CI)	AUPRC (95% CI)	Sensitivity (95% CI)	Specificity (95% CI)	F1	Accuracy
BreathGOUT	Extra Trees	451	0.391	0.999 (0.997–1.000)	0.999 (0.997–1.000)	0.990 (0.967–1.000)	0.990 (0.966–1.000)	0.990	0.990
BreathRiskGOUT	Extra Trees	456	0.391	0.999 (0.997–1.000)	0.999 (0.997–1.000)	0.990 (0.967–1.000)	0.990 (0.966–1.000)	0.990	0.990
BreathGOUT-Plus	Extra Trees	484	0.391	0.999 (0.997–1.000)	0.999 (0.997–1.000)	0.990 (0.967–1.000)	0.990 (0.966–1.000)	0.990	0.990
RiskGOUT	Logistic Regression	5	0.384	0.890 (0.840–0.930)	0.878 (0.807–0.933)	0.909 (0.848–0.960)	0.716 (0.620–0.798)	0.826	0.811
TongueGOUT	Extra Trees	28	0.481	0.654 (0.578–0.728)	0.667 (0.571–0.760)	0.657 (0.563–0.748)	0.588 (0.490–0.683)	0.631	0.622

### 3.4 Model construction and validation

To systematically evaluate the contribution of each modality, we developed diagnostic models on five feature configurations: breath metabolite markers alone (BreathGOUT), clinical characteristic alone (RiskGOUT), tongue-image features alone (TongueGOUT), and their combinations (BreathRiskGOUT and BreathGOUT-Plus). All models were trained and evaluated using the identical machine-learning pipeline and 5-fold stratified cross-validation protocol.

First, three machine-learning models were trained on the full set of 451 breath molecular features, with the top 30 features selected within each training fold; the 12 differential features identified by global screening were evaluated separately as candidate biomarkers and were not used as model input. As shown in Table 4, the Extra Trees model performed best and was selected as BreathGOUT, achieving an AUC of 0.999 (95% CI 0.997–1.000) for the 451-feature input with per-fold top-30 selection, an accuracy of 0.990, a sensitivity of 0.990 (95% CI 0.967–1.000), and a specificity of 0.990 (95% CI 0.966–1.000). Logistic regression and random forest also achieved very high AUC values (0.997 and 0.998, respectively), indicating that the discriminative signal was not entirely dependent on a single complex model, but rather suggested considerable intergroup separation inherent in the breath metabolite features themselves. (Figure 8).

**FIGURE 8:**
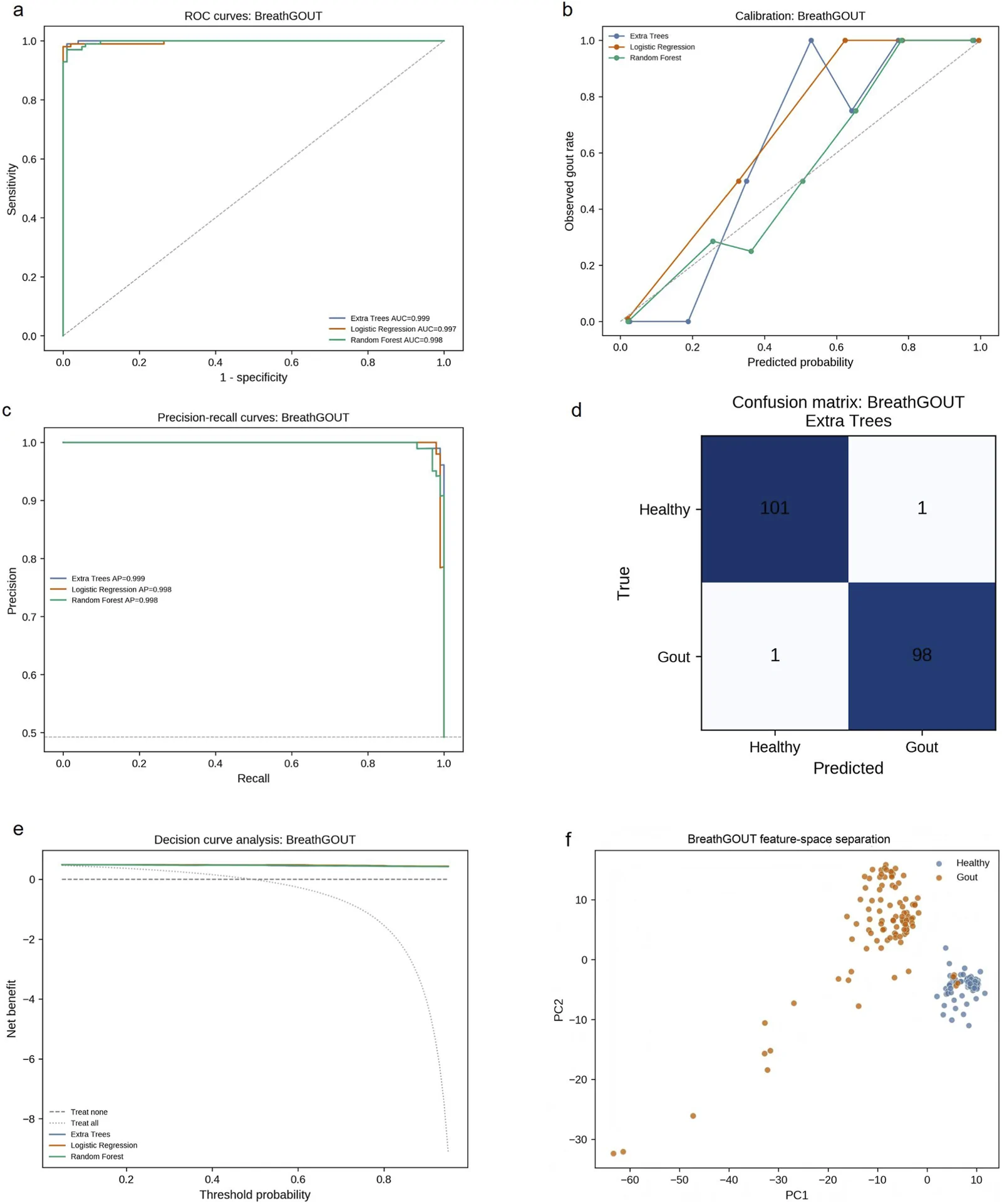
Integrated performance evaluation of the BreathGOUT gout diagnostic model based on exhaled breath molecular features. **(a)** Receiver operating characteristic curves of the extreme randomized tree, logistic regression, and random forest algorithms for discriminating patients with gout from healthy controls; **(b)** calibration curves showing agreement between predicted probabilities and observed gout prevalence; **(c)** precision–recall curves and average precision values for the three machine-learning models; **(d)** Confusion matrix of the optimal extreme randomized tree model, showing the numbers of true positives, true negatives, false positives, and false negatives. Predictions were aggregated from out-of-fold predictions across 5-fold stratified cross-validation (n = 201); **(e)** decision curve analysis comparing the three models with two reference strategies (treat-none and treat-all); and **(f)** two-dimensional principal component analysis scatter plot based on BreathGOUT breath molecular features, illustrating separation between the gout group (orange circles) and healthy participants (blue circles) in feature space.

The BreathGOUT-Plus model was developed by integrating the BreathGOUT features with conventional clinical characteristics and tongue features (Supplementary Figures 11–15). The best-performing combined model was also Extra Trees, with an AUC of 0.999 (95% CI 0.997–1.000), a sensitivity of 0.990, and a specificity of 0.990; the intermediate BreathRiskGOUT configuration performed equivalently (Table 4; Supplementary Figures 16–20). These results indicate that BreathGOUT-Plus did not further improve model performance relative to BreathGOUT, suggesting that the dominant diagnostic information in the present dataset was derived from breath mass-spectrometry data.

To directly evaluate whether the global signal-intensity difference between groups could drive feature selection and model performance, we performed four analyses (Supplementary Figure 21). First, total ion current (TIC) differed markedly between groups (healthy: median 3.58 × 10^7^, IQR 2.67–3.88 × 10^7^; gout: median 1.57 × 10^6^, IQR 1.07–2.39 × 10^6^; P = 2.0 × 10^−32^), and TIC alone discriminated between groups with a cross-validated AUC of 0.984 (logistic regression) to 0.988 (Extra Trees). Second, BreathGOUT cross-validated predictions correlated with TIC across all subjects (Spearman r = −0.868), although within-group correlations were considerably weaker (healthy r = −0.565; gout r = −0.343). Third, and most importantly, classification performance was essentially unchanged across preprocessing strategies, with 5-fold cross-validated AUCs of 0.999 for TIC normalization, 0.995 for median normalization, 0.999 for probabilistic quotient normalization, and 0.998 when no normalization was applied, indicating that discriminative information resides in the relative-abundance pattern of the mass spectra rather than in global signal intensity, which is mathematically removed by per-sample normalization. Together, these analyses indicate that the near-perfect classification performance of BreathGOUT is not driven by the global signal-intensity difference between groups.

To distinguish breath-specific metabolic signals from potential confounding by baseline clinical characteristics (Table 1), we next evaluated models trained on each non-breath modality alone. RiskGOUT, comprising 5 clinical variables (age, BMI, smoking history, drinking history, and family history), achieved moderate discrimination, with logistic regression as the best model reaching an AUC of 0.890 (95% CI 0.840–0.930), a sensitivity of 0.909 (95% CI 0.848–0.960), and a specificity of 0.716 (95% CI 0.620–0.798) (Figures 9A–E). TongueGOUT, comprising 28 tongue-image features, showed limited discriminative ability, with Extra Trees as the best model achieving an AUC of 0.654 (95% CI 0.578–0.728), a sensitivity of 0.657 (95% CI 0.563–0.748), and a specificity of 0.588 (95% CI 0.490–0.683) (Figures 9F–J).

**FIGURE 9:**
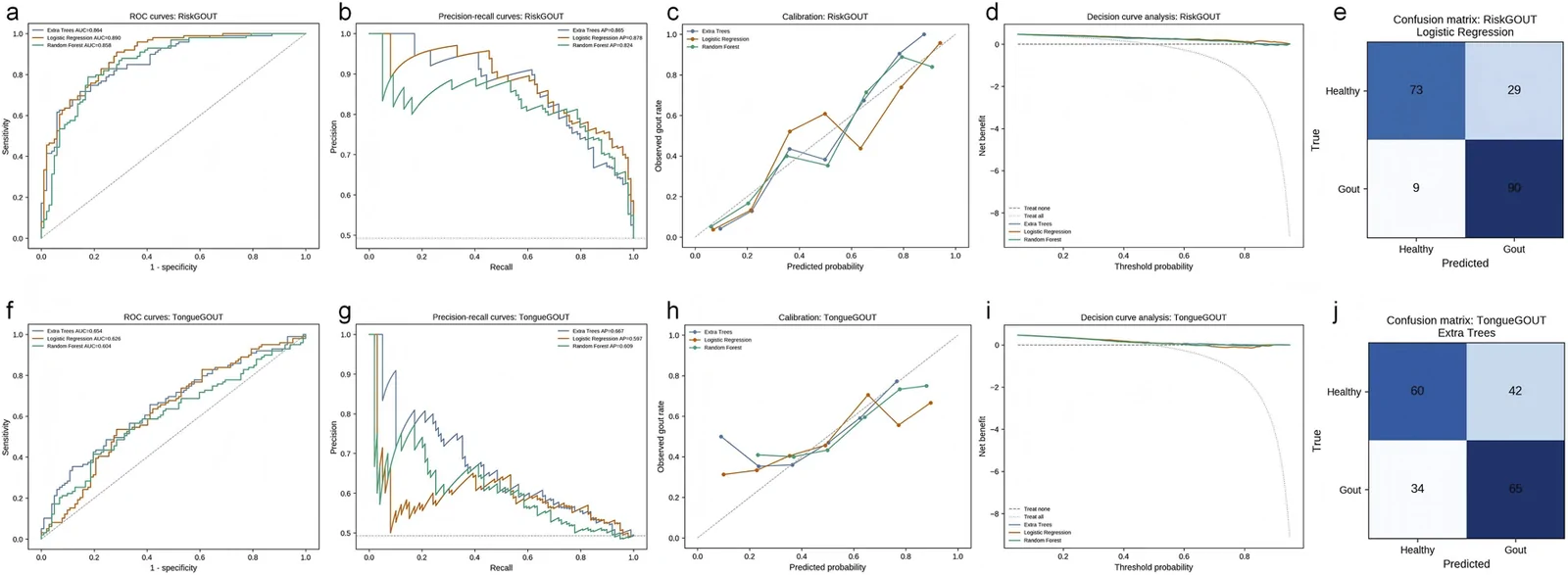
Integrated performance evaluation of the RiskGOUT and TongueGOUT unimodal control models. **(a)** Receiver operating characteristic curves of the extreme randomized tree, logistic regression, and random forest algorithms for the RiskGOUT model in discriminating patients with gout from healthy controls; **(b)** precision–recall curves and average precision values for the three machine-learning models in RiskGOUT; **(c)** calibration curves showing agreement between predicted probabilities and observed gout prevalence in RiskGOUT; **(d)** confusion matrix of the optimal logistic regression model for RiskGOUT, showing the numbers of true positives, true negatives, false positives, and false negatives; **(e)** decision curve analysis comparing the three RiskGOUT models with two reference strategies (treat-none and treat-all); **(f)** receiver operating characteristic curves of the three algorithms for the TongueGOUT model; **(g)** precision–recall curves and average precision values for TongueGOUT; **(h)** calibration curves for TongueGOUT; **(i)** confusion matrix of the optimal extreme randomized tree model for TongueGOUT **(j)** decision curve analysis comparing the three TongueGOUT models with the two reference strategies. Predictions were aggregated from out-of-fold predictions across 5-fold stratified cross-validation (n = 201).

Taken together, the five feature configurations formed a clear three-tier gradient of discrimination (Figure 10): breath-based models consistently achieved AUCs of 0.997–0.999, models based on clinical characteristics alone reached a mean AUC of 0.871, and models based on tongue images alone reached a mean AUC of 0.628, underscoring that the diagnostic signal in this cohort originates predominantly from breath metabolite features.

**FIGURE 10:**
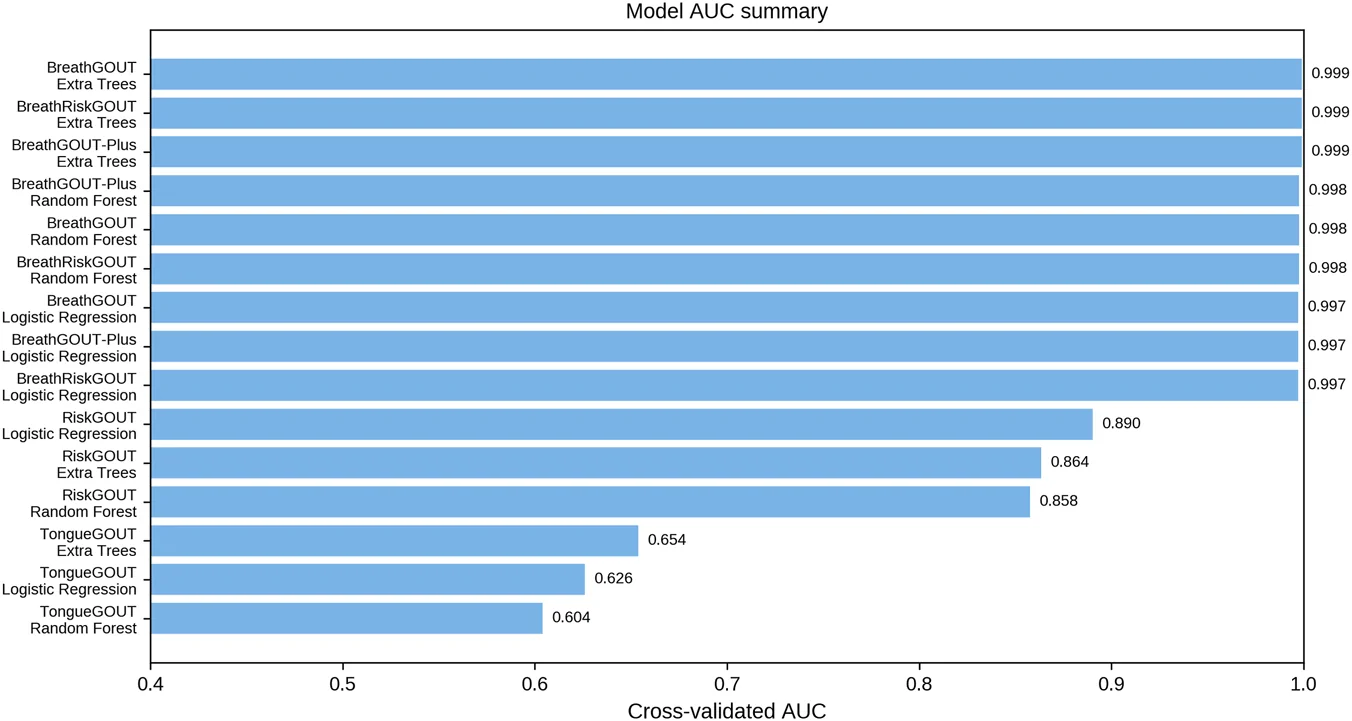
AUC values across all data-driven models. The model incorporating exhaled breath molecular features achieved the best overall performance, whereas TongueGOUT performed relatively less well.

## 4 Discussion

Breath molecular feature-based diagnostic models offer several clear advantages, including simple sampling, rapid real-time detection, noninvasiveness, and the feasibility of repeated measurements for dynamic monitoring (Miao et al., 2023). In the present study, we found that breath molecular features analyzed by machine-learning algorithms showed promising discriminatory capacity between gout patients and healthy controls. By contrast, conventional diagnostic approaches remain limited in their sensitivity for early-stage disease, whereas our model provides a promising noninvasive alternative. Among the tested algorithms, the decision tree–based model achieved the best performance, with an AUC exceeding 0.90, indicating that breath molecular features capture substantial metabolic alterations associated with gout. A key strength of this study lies in the robustness of the breath molecular feature signal; therefore, the identified breath molecular features are likely to reflect genuine disease-related metabolic perturbations rather than random noise. Nevertheless, these findings should be interpreted cautiously because this was a preliminary single-center study with a limited sample size.

An increasing body of evidence indicates that volatile organic compounds can serve as biomarkers for metabolic diseases such as diabetes (Wang et al., 2021), diabetic nephropathy (Kreepala et al., 2025), obesity, and metabolic syndrome (Bosch-Sierra et al., 2025). Several studies have also investigated the diagnostic value of VOCs in gout, and our findings are broadly consistent with this growing literature. For example, Li et al. analyzed urine samples from volunteers using GC-MS and identified 30 differential VOCs, among which urate and isoxanthopterin were proposed as potential biomarkers for gout diagnosis (Zhang and Liu, 2018). Zhao et al. further demonstrated in an epidemiological study that accumulation of phthalate-related VOCs in urine may induce systemic low-grade inflammation and increase serum urate levels, representing an environmental risk factor for hyperuricemia and gout (Zhao and Li, 2025). This evidence reinforces the link between VOCs, disordered purine metabolism, and inflammatory activation. Similarly, Yun et al. used ultra-performance liquid chromatography–mass spectrometry (UPLC-MS) and identified pyroglutamic acid, 2-methylbutyl carnitine, and Phe-Phe as major biomarkers for distinguishing patients with gout from healthy volunteers (Huang et al., 2020). These studies provide important clues regarding purine metabolic dysregulation in gout and support the plausibility of our identification of inosine and urate dimer as gout-associated markers in exhaled breath (Li Y. et al., 2023; Jia et al., 2025). From a technical perspective, GC-MS remains the reference standard for breath VOC analysis because of its high specificity and sensitivity (Bajo-Fernández et al., 2024). However, its high cost, procedural complexity, and lack of portability limit its applicability in large-scale screening. By contrast, real-time online techniques such as neutral desorption electrospray extraction ionization mass spectrometry (ND-EESI-MS) (Zheng et al., 2021) and high-resolution electrospray extraction ionization mass spectrometry (EESI-MS) (Li et al., 2013) have attracted increasing attention because of their high-throughput capability, and some platforms are already progressing toward commercialization, including VOCs-based breath analyzers (Moura et al., 2023; Pathak et al., 2023).

The first major challenge in interpreting our findings is determining the true nature of the signal captured by the BreathGOUT model. Although BreathGOUT achieved an AUC of 0.999 (95% CI 0.997–1.000), this apparently outstanding performance must be assessed in the context of baseline characteristic imbalance. Patients with gout and healthy controls differed significantly in BMI (27.88 ± 4.64 vs. 22.07 ± 3.44, p < 0.001), alcohol consumption history (65.7% vs. 32.4%, p < 0.001), and smoking history (44.4% vs. 26.5%, p = 0.012) (Table 1), suggesting a potential risk of phenotypic confounding. As shown in Table 4 and Figure 9, the unimodal decomposition analysis revealed a clear three-tier gradient of discrimination, with mean AUCs of 0.871 for RiskGOUT, 0.628 for TongueGOUT, and 0.999 for BreathGOUT. This gradient is informative: if the performance of BreathGOUT were primarily driven by clinical variables, then RiskGOUT should have approached 0.999. Instead, its mean AUC was only 0.871, representing a substantial gap of 0.128 from BreathGOUT. Because an AUC difference greater than 0.05 is considered clinically meaningful in diagnostic testing (Xu et al., 2020), these data indicate that clinical characteristics accounted for only part of the between-group separability, whereas the breath metabolite-derived signal contributed the remaining disease-specific information that elevated discrimination to such a promising level in this single-center case-control cohort. In other words, breath molecular features likely capture a metabolic dimension that is not accessible through conventional phenotype-based risk assessment. From a mechanistic perspective, BMI and alcohol history reflect chronic behavioral exposure, whereas breath molecular features may capture metabolic processes related to purine handling that are not fully represented by BMI or alcohol history. The upregulation of the putative urate dimer (m/z 318.0) is consistent with increased urate burden; however, given its unconfirmed structure and the absence of longitudinal data, it should not be interpreted as a marker of crystallization risk at this stage. Hyperuricemic states caused by defects in purine metabolism enzymes are not reversed by weight reduction, and the urate dimer signal may persist in breath despite changes in body habitus. This partial decoupling of molecular phenotype from clinical phenotype may contribute to the discriminatory information captured by breath molecular features beyond clinical characteristics. We emphasize, however, that the present data do not establish independence from clinical confounding: BMI, alcohol consumption, and smoking may themselves influence the breath metabolic profile, and BMI-stratified or propensity-score-matched analyses were not performed.

On the basis of differential feature analysis, we identified 12 breath molecular features with high discriminatory performance. Of these, only the ion at m/z 318.0 was upregulated, whereas the remaining 11 ions were downregulated. The increase in m/z 318.0 is consistent with the putative urate dimer, which may reflect urate accumulation, although tandem MS confirmation is needed. The coordinated downregulation of the other 11 ions is intriguing, but these identifications remain putative, and their biological interpretation is speculative without confirmed structures or complementary functional data. Among numerous possible explanations, these patterns could conceivably relate to metabolic alterations in lipid, mitochondrial, or microbial pathways—categories tentatively suggested by m/z matching against published databases—but we emphasize that these associations are unvalidated and that alternative explanations cannot be excluded. Future studies incorporating structural confirmation, isotope tracing, and targeted metabolomics will be required to explore these observations. (Figure 11).

**FIGURE 11:**
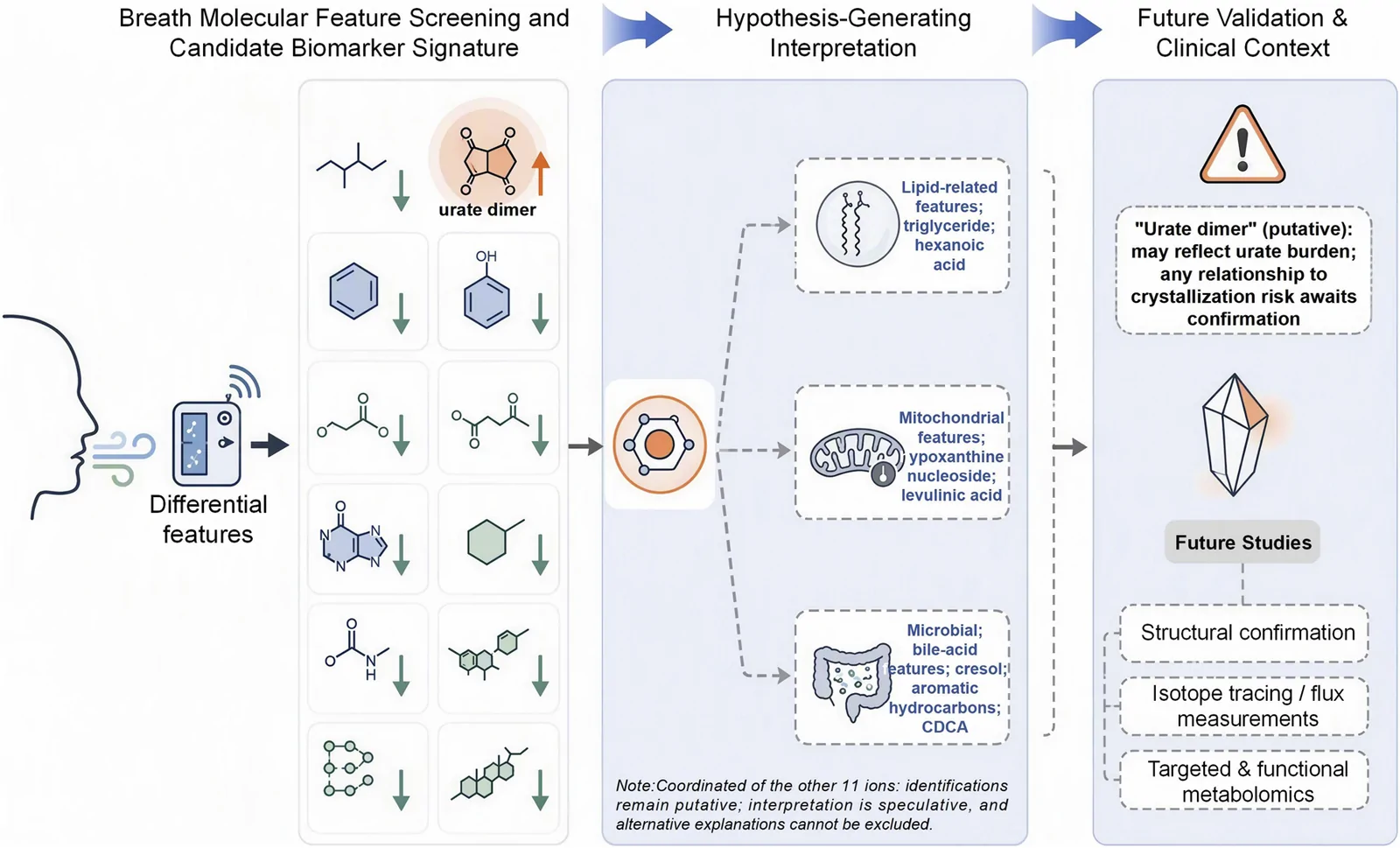
Schematic overview of breath molecular features screening and hypothesis-generating interpretation.Breath metabolomics identified 12 discriminatory breath molecular features: m/z 318.0 (putative urate dimer) was upregulated and the other 11 ions downregulated. The m/z 318.0 increase suggests putative urate accumulation; the 11 downregulated ions were tentatively grouped into lipid-, mitochondrial-, and microbial/bile-acid–related features by database m/z matching. All identifications are putative (dashed lines = unvalidated, hypothesis-generating associations). The putative urate dimer may reflect urate burden; whether it relates to crystallization risk remains unknown and requires structural confirmation and longitudinal studies.

We further developed a three-modality diagnostic model, BreathGOUT-Plus, integrating breath molecular features, clinical characteristics, and tongue-image features to improve the clinical utility of gout diagnosis in this single-center case-control cohort. Internal cross-validation indicated that the bimodal BreathRiskGOUT model and the trimodal fused model achieved AUC values ranging from 0.997 to 0.999, with no statistically significant difference from the unimodal BreathGOUT model, indicating convergent discriminative performance across the three models. The mean AUC of the model based on breath molecular features alone reached 0.998, demonstrating that the breath molecular features of patients with gout and healthy participants in this cohort showed substantial distributional separation, demonstrating promising discrimination in this single-center case-control cohort on the basis of the breath metabolic fingerprint alone. Under this performance ceiling, additional clinical and tongue-image modalities contributed only minimal marginal information and did not improve quantitative diagnostic accuracy. This does not mean that the auxiliary modalities lack biological relevance; rather, it indicates that the breath molecular features already captured a large proportion of effective stratification information in this cohort. Specifically, the clinical risk-factor model achieved an AUC of 0.871, and its stratification information appeared to be largely captured by the breath molecular features, whereas the tongue-image model performed even less well and provided no meaningful incremental value.

Although multimodal fusion did not increase the AUC, it remains of distinct clinical and research value. Its main advantage lies not in improving classification performance *per se*, but in strengthening the logic of integrated clinical diagnosis (Park and Lee, 2025). In principle, multimodal integration may improve clinical robustness. Breath molecular features are susceptible to transient external influences such as recent high-purine intake, alcohol consumption, and urate-lowering medications, which may induce short-lived signal fluctuations. By contrast, clinical characteristic such as BMI and family history of gout are stable intrinsic characteristics. When breath molecular feature signals are perturbed by external factors, clinical baseline variables may serve as a stable reference and help reduce false-negative classification. We therefore hypothesize that even if BreathRiskGOUT and BreathGOUT exhibited similar AUC values, the fused model may provide a broader decision boundary and improved tolerance to atypical samples. In addition, the multimodal framework enables cross-validation across different dimensions of risk. When the predicted risk differs across modalities, the discordance itself becomes an independent clinical clue: if the breath molecular feature model indicates high risk but clinical variables and tongue features are unremarkable, the elevated breath molecular feature signal may be attributable to short-term dietary or alcohol-related perturbations before sampling; conversely, if traditional clinical characteristic indicate high risk but breath molecular features remain unremarkable, this may suggest a compensated stage of purine metabolism or effective urate-lowering treatment. These interpretations are hypothesis-generating and require prospective validation. Moreover, the synergy between tongue features and breath molecular features may help build a link between traditional Chinese medicine syndrome patterns and purine metabolic dysregulation, thereby improving interpretability and integrating Western and Eastern diagnostic dimensions, which is a benefit unattainable with unimodal models.

The relatively low AUC of the tongue-coating model alone, ranging from 0.604 to 0.654, should be interpreted from both technical and pathological perspectives. From a technical standpoint, RGB-based chromatic features are potentially sensitive to illumination color temperature and camera distance. Although image acquisition was standardized in this study, camera-to-tongue distance and tongue-protrusion duration were not quantitatively logged and may still have introduced residual variability into chromatic measurements. In addition, the duration of tongue protrusion may alter microcirculatory perfusion of the tongue and confound quantitative redness measurements, and this variable was not incorporated into quality control. Furthermore, current tongue-image AI systems typically use deep architectures such as ResNet and Vision Transformer for end-to-end feature extraction, whereas our study relied on handcrafted RGB/HSV features; this methodological gap likely contributed to reduced stability. From a pathological standpoint, the core lesion in gout is dysregulation of the purine–urate metabolic pathway, whereas abnormal tongue coating is usually a later accompanying sign of microcirculatory disturbance, meaning that its diagnostic window lags behind the molecular changes captured by breath molecular features. The TCM “damp-heat” pattern is also not disease-specific, as yellow greasy tongue coating can be observed in obesity, diabetes, and chronic kidney disease; therefore, syndrome-based indicators have an inherent upper limit in independently distinguishing gout. Nevertheless, tongue features remain valuable within a multimodal framework. A high BreathGOUT score with normal tongue findings suggests that the elevated breath molecular feature signal may reflect short-term dietary perturbation, whereas a yellow greasy tongue coating with only a moderate BreathGOUT score may indicate subclinical inflammation or early metabolic abnormality. This temporal mismatch between syndrome features and metabolic changes is precisely the deeper advantage of multimodal architecture.

Several limitations of this study should be stated objectively in terms of evidence level, methodological rigor, and technical standardization. First, this was a single-center study with a modest sample size (n = 201), which limits generalizability. The key biomarker m/z 318.0 (urate dimer) was tentatively identified and was not confirmed using authentic standards or MS/MS fragmentation; however, this limitation reflects the broader challenge of incomplete metabolomics database coverage rather than an intrinsic flaw of EESI-MS. Future work should improve identification confidence through synthetic standard matching and targeted MS/MS validation. Second, although unimodal decomposition analysis was used to exclude baseline confounding as the main driver of the high AUC of the breath molecular feature model, strict stratified sensitivity analyses, such as BMI stratification or propensity score matching, were not performed. This omission was not deliberate but was constrained by limited sample size and insufficient statistical power; such analyses should be incorporated in larger cohorts. Third, although illumination was standardized at 5000 K and 4,800 lx with fixed camera settings, camera distance and tongue-protrusion duration were not quantitatively recorded, which may have introduced residual variability into the “redness” metric. Although these technical issues do not alter the direction of the present conclusions, they may limit cross-center implementation. Fourth, renal function was not systematically assessed at baseline, which limits our ability to evaluate potential confounding by renal function and to fully characterize the relationship between breath molecular features and urate metabolism. Future studies will incorporate renal function assessment alongside breath sampling to clarify these relationships. Fifth, a marked global difference in total signal intensity was observed between groups. Although classification performance was robust to alternative normalization strategies, the biological or technical origin of this intensity difference remains unclear; future studies should therefore adopt interleaved case-control acquisition, internal standards, and continuous instrument-drift monitoring to clarify its nature.

Based on these limitations, future studies should proceed in four directions. First, multicenter, large-scale prospective cohorts should be established, incorporating BMI-stratified and propensity-score-matched analyses to exclude residual confounding and confirm generalizability. Second, synthetic standard matching and targeted MS/MS should be pursued to confirm the putative urate dimer (m/z 318.0) and upgrade biomarker annotation confidence. Third, renal function should be systematically assessed alongside breath sampling in future studies, enabling direct correlation of breath molecular features with renal urate handling; within this framework, longitudinal tracking of hyperuricemic individuals—both before and after urate-lowering therapy—would extend BreathGOUT from diagnosis to prediction and treatment monitoring. Finally, tongue-image acquisition should include color-temperature calibration, and model architecture should evolve toward attention-based adaptive fusion, shifting weight dynamically between the breath molecular feature and clinical modalities to achieve clinical robustness.

## 5 Conclusion

In summary, in this single-center proof-of-concept study, machine-learning analysis of the breath molecular features accurately discriminated established gout from healthy states and identified 12 candidate ions as putative biomarkers, with discriminatory performance largely complementary to, though not yet demonstrably independent of, conventional clinical characteristics. However, the modest, single-center, case-control cohort, the putative nature of the molecular annotations, and the absence of external validation limit the generalizability of these findings. Multicenter, prospective studies encompassing asymptomatic hyperuricemia, first-presentation arthritis, and other arthritides are warranted to establish the translational potential of breath metabolomics for the early detection and management of gout.

## Data Availability

The original contributions presented in the study are included in the article/Supplementary Material, further inquiries can be directed to the corresponding authors.

## References

[B1] Azim A. Barber C. Dennison P. Riley J. Howarth P. (2019). Exhaled volatile organic compounds in adult asthma: a systematic review. Eur. Respir. J. 54 (3), 1900056. 10.1183/13993003.00056-2019 31273044

[B2] Bajo-Fernández M. Souza-Silva É. A. Barbas C. Rey-Stolle M. F. García A. (2024). GC-MS-Based metabolomics of volatile organic compounds in exhaled breath: applications in health and disease. A review. Front. Mol. Biosci. 10, 1295955. 10.3389/fmolb.2023.1295955 38298553 PMC10828970

[B3] Bosch-Sierra N. Navajas-Porras B. Grau-Del Valle C. Salom C. Pérez-Burillo S. D'Auria G. (2025). Effect of a multiphase dietary intervention on metabolic profile and gut microbiota composition in metabolically healthy and unhealthy obese subjects. Clin. Nutr. 54, 62–70. 10.1016/j.clnu.2025.09.010 41033075

[B4] Cen Z. Huang Y. Li S. Dong S. Wang W. Li X. (2024). Advancing breathomics through accurate discrimination of endogenous from exogenous volatiles in breath. Environ. Sci. Technol. 58 (42), 18541–18553. 10.1021/acs.est.4c04575 39340814

[B5] Chinese Society of Endocrinology, Chinese Medical Association (2020). Clinical practice guideline for the management of hyperuricemia and gout in China (2019). Chin. J. Endocrinol. Metab. 36 (1), 1–13. 10.3760/cma.j.issn.1000-6699.2020.01.001

[B6] Chou H. Godbeer L. Allsworth M. Boyle B. Ball M. L. (2024). Progress and challenges of developing volatile metabolites from exhaled breath as a biomarker platform. Metabolomics 20 (4), 72. 10.1007/s11306-024-02142-x 38977623 PMC11230972

[B7] Dalbeth N. Gosling A. L. Gaffo A. Abhishek A. (2021). Gout. Lancet 397 (10287), 1843–1855. 10.1016/S0140-6736(21)00569-9 33798500

[B8] FitzGerald J. D. (2025). Gout. Ann. Intern Med. 178 (3), ITC33–ITC48. 10.7326/ANNALS-24-03951 40063960

[B9] Hofstadler S. A. Sannes-Lowery K. A. (2006). Applications of ESI-MS in drug discovery: interrogation of noncovalent complexes. Nat. Rev. Drug Discov. 5 (7), 585–595. 10.1038/nrd2083 16816839

[B10] Hu Y. Niu Z. Cao C. Gao J. Pan M. Cai Y. (2024). Volatile organic compounds (VOC) VOCs in urine are associated with increased systemic inflammation levels, and smokers are identified as a vulnerable population. Ecotoxicol. Environ. Saf. 288, 117398. 10.1016/j.ecoenv.2024.117398 39612684

[B11] Huang Y. Xiao M. Ou J. Lv Q. Wei Q. Chen Z. (2020). Identification of the urine and serum metabolomics signature of gout. Rheumatol. Oxf. 59 (10), 2960–2969. 10.1093/rheumatology/keaa018 32134107

[B12] Jia Q. Dong Q. Zhang J. Zhao Q. Li Y. Chao Z. (2025). Untargeted metabolomics analysis of the urinary metabolic signature of acute and chronic gout. Clin. Chim. Acta 565, 119968. 10.1016/j.cca.2024.119968 39276825

[B13] Ke M. Zhang H. Ding J. Xiong X. Li F. Chingin K. (2019). Generating supercharged protein ions for breath analysis by extractive electrospray ionization mass spectrometry. Anal. Chem. 91 (5), 3215–3220. 10.1021/acs.analchem.8b03114 30707562

[B14] Keller S. F. Mandell B. F. (2022). Management and cure of gouty arthritis. Rheum. Dis. Clin. North Am. 48 (2), 479–492. 10.1016/j.rdc.2022.03.001 35400373

[B15] Kreepala C. Ayutaya V. S. N. Phatthanakun R. Thabsuwan K. Paewponsong J. (2025). The biosensor using the target urine volatile organic compounds for detecting diabetic kidney disease. Sci. Rep. 15 (1), 14738. 10.1038/s41598-025-00013-6 40289139 PMC12034762

[B16] Kuo C. F. Grainge M. J. Zhang W. Doherty M. (2015). Global epidemiology of gout: prevalence, incidence and risk factors. Nat. Rev. Rheumatol. 11 (11), 649–662. 10.1038/nrrheum.2015.91 26150127

[B17] Kuo T. C. Tan C. E. Wang S. Y. Lin O. A. Su B. H. Hsu M. T. (2020). Human breathomics database. Database (Oxford) 2020, baz139. 10.1093/database/baz139 31976536 PMC6978997

[B18] Li M. Hu B. Li J. Chen R. Zhang X. Chen H. (2009). Extractive electrospray ionization mass spectrometry toward *in situ* analysis without sample pretreatment. Anal. Chem. 81 (18), 7724–7731. 10.1021/ac901199w 19673501

[B19] Li P. Deng L. Luo J. (2013). EESI-MS detection and statistical analysis of multi-batch of exhaled breath metabolomics data of liver failure patients. Chem. J. Chin. Univ. 34 (5), 1105–1110. 10.7503/cjcu20120832

[B20] Li R. Liang N. Tao Y. Yin H. (2023a). Metabolomics in hyperuricemia and gout. Gout Urate Cryst. Depos. Dis. 1 (1), 49–61. 10.3390/gucdd1010006

[B21] Li Y. Han X. Tong J. Wang Y. Liu X. Liao Z. (2023b). Analysis of VOCs in gout: a systematic review and meta-analysis. Nutrients 15 (14), 3143. 10.3390/nu15143143 PMC10383779 37513561

[B22] Lu G. Ji T. He S. Ai F. Yan L. Hu J. (2025). Recent progress of exhaled gas-based diagnosis based on field effect transistor sensors. Adv. Funct. Mater 35 (21), 2309111. 10.1002/adfm.202309111

[B23] Miao G. Wang Y. Wang B. Yu H. Liu J. Pan R. (2023). Multi-omics analysis reveals hepatic lipid metabolism profiles and serum lipid biomarkers upon indoor relevant VOC exposure. Environ. Int. 180, 108221. 10.1016/j.envint.2023.108221 37742460

[B24] Moura P. C. Raposo M. Vassilenko V. (2023). Breath volatile organic compounds (VOCs) as biomarkers for the diagnosis of pathological conditions: a review. Biomed. J. 46 (4), 100623. 10.1016/j.bj.2023.100623 37336362 PMC10339195

[B25] Neogi T. Jansen T. L. Dalbeth N. Fransen J. Schumacher H. R. Berendsen D. (2015). Gout classification criteria: an American college of rheumatology/european league against rheumatism collaborative initiative. Arthritis Rheumatol. 67 (10), 2557–2568. 10.1002/art.39254 26352873 PMC4566153

[B26] Park J. Lee S. (2025). MoltiTox: a multimodal fusion model for molecular toxicity prediction. Front. Toxicol. 7, 1720651. 10.3389/ftox.2025.1720651 41488760 PMC12756560

[B27] Pathak A. K. Swargiary K. Kongsawang N. Jitpratak P. Ajchareeyasoontorn N. Udomkittivorakul J. (2023). Recent advances in sensing materials targeting clinical volatile organic compound (VOC) biomarkers: a review. Biosensors 13 (1), 114. 10.3390/bios13010114 36671949 PMC9855562

[B28] Qin M. Qian Y. Huang L. Zhong C. Li M. Yu J. (2023). Extractive electrospray ionization mass spectrometry for analytical evaluation and synthetic preparation of pharmaceutical chemicals. Front. Pharmacol. 14, 1110900. 10.3389/fphar.2023.1110900 36713836 PMC9880169

[B29] Singh J. A. Gaffo A. (2020). Gout epidemiology and comorbidities. Semin. Arthritis Rheum. 50 (3S), S11–S16. 10.1016/j.semarthrit.2020.04.008 32620196

[B30] van Vorstenbosch R. van Munster K. Stavropoulos G. Pachen D. van Schooten F. J. Ponsioen C. (2024). The potential of volatile organic compounds to diagnose primary sclerosing cholangitis. JHEP Rep. 6 (8), 101103. 10.1016/j.jhepr.2024.101103 39131082 PMC11315128

[B31] Wang W. Zhou W. Wang S. Huang J. Le Y. Nie S. (2021). Accuracy of breath test for diabetes mellitus diagnosis: a systematic review and meta-analysis. BMJ Open Diabetes Res. Care 9 (1), e002174. 10.1136/bmjdrc-2021-002174 34031142 PMC8149324

[B32] Wei Y. Qiu X. Su L. Tang X. Chen Y. Liu S. (2026). A machine learning approach to metabolomics identifies putative biomarker candidates and dysregulated pathways for distinguishing gout from asymptomatic hyperuricemia in the Zhuang population. Metabolomics 22 (4), 124. 10.1007/s11306-026-02501-w 42435238 PMC13356051

[B33] Xu T. Huang C. Liu Y. Gao J. Chang H. Yang R. (2020). Artificial intelligence based on deep learning for differential diagnosis between benign and malignant pulmonary nodules: a real-world, multicenter, diagnostic study. J. Clin. Oncol. 38 (15), 9037. 10.1200/jco.2020.38.15_suppl.9037

[B34] Zhang L. Liu Y. (2018). Urinary metabolomics study of patients with gout using gas chromatography-mass spectrometry. J. Anal. Methods Chem. 2018, 3461572. 10.1155/2018/3461572 PMC6206583 30410926

[B35] Zhang X. Han Y. N. Cao T. Zhang J. Zhang Y. Liu Z. (2025). Breath volatile organic compounds as non-invasive tools for the detection and monitoring of inflammatory bowel disease. J. Gastroenterol. Hepatol. 40 (10), 2482–2491. 10.1111/jgh.70061 40866317

[B36] Zhao Z. H. Li M. (2025). Urinary volatile organic compound VOCs correlate with systemic inflammation and hyperuricemia risk in US adults. Environ. Int. 198, 108967. 10.1016/j.envint.2025.108967

[B37] Zheng Q. Zhang J. Wang X. Zhang W. Xiao Y. Hu S. (2021). Neutral desorption extractive electrospray ionization mass spectrometry analysis sputum for non-invasive lung adenocarcinoma detection. Onco Targets Ther. 14, 469–479. 10.2147/OTT.S269300 33488101 PMC7816046

[B38] Zhu Y. Pandya B. J. Choi H. K. (2012). Comorbidities of gout and hyperuricemia in the US general population: NHANES 2007–2008. Am. J. Med. 125 (7), 679–687.e1. 10.1016/j.amjmed.2011.09.033 22626509

