# Supplementary material for: Breath molecular feature profiling with machine learning for noninvasive diagnosis of gout: a single-center case-control study
Source: Front Mol Biosci. 2026 Oct 21;13:1939939. doi: 10.3389/fmolb.2026.1939939 (PMC13730828; doi:10.3389/fmolb.2026.1939939)
Supplement: Supplementary file 1 [file Table1.docx]

Supplementary Material

**I. Appendix BreathGOUT-Plus**

This multimodal model integrates exhaled breath VOCs, tongue manifestation images and clinical risk factors. The optimal performance was achieved by the Extra Trees algorithm, with an AUC of 0.999, sensitivity of 0.990 and specificity of 0.990.

Figure 11. ROC curve of the BreathGOUT-Plus model. The multimodal combined model retains high discriminative performance comparable to the breath-only model.

Figure 12. Precision-Recall curve of the BreathGOUT-Plus model.

Figure 13. Calibration curve of the BreathGOUT-Plus model.

Figure 14. Confusion matrix of the optimal BreathGOUT-Plus model.

Figure 15. Decision curve analysis of the BreathGOUT-Plus model.

II. Appendix BreathRiskGOUT

Figure 16. ROC curve of BreathRiskGOUT.

Figure 17. Precision-Recall curve of BreathRiskGOUT.

Figure 18. Calibration curve of BreathRiskGOUT.

Figure 19. Confusion matrix of the optimal BreathRiskGOUT model.

Figure 20. Decision curve analysis of BreathRiskGOUT.

III. Analytical assessment of the potential contribution of global signal-intensity differences to model performance.

Figure 21.(A) Distribution of total ion current (TIC) per sample in healthy controls (n = 102) and gout patients (n = 99); (B) receiver operating characteristic curve of TIC alone (5-fold cross-validation); (C) relationship between BreathGOUT cross-validated predicted probability and TIC (Spearman r = −0.868 overall; r = −0.565 in healthy and r = −0.343 in gout); (D) cross-validated AUC of the BreathGOUT model under alternative preprocessing strategies (TIC normalization, no normalization, median normalization, and probabilistic quotient normalization) and of models using TIC alone. All models used Extra Trees with the top 30 features selected within each training fold (5-fold stratified cross-validation).

IV. Appendix Supplementary Table 1：

Supplementary Table 1. Data dictionary of model configurations and cross-validation design.

| **Model** | **Candidate variables (n)** | **Retained after selection (n)** | **Final variable names** | **Algorithm** | **Training n**  **(per fold)** | **Test n**  **(per fold)** |
| --- | --- | --- | --- | --- | --- | --- |
| BreathGOUT | 451 | 30 | Top 30 m/z ions (50-500 Da) selected via fold-wise SelectKBest (f_classif) | Logistic Regression / Random Forest / Extra Trees | ~161 | ~40 |
| RiskGOUT | 5 | 5 | Age; BMI; Smoking history; Alcohol consumption; Family history of gout | Logistic Regression / Random Forest / Extra Trees | ~161 | ~40 |
| TongueGOUT | 28 | 28 | 28 aggregated tongue-image features (14 tongue-body + 14 sublingual): tongue_mask_ratio,R_mean,G_mean,B_mean,R_std,G_std,B_std,redness_index, yellow_coating_index, whiteness_index, darkness_index, saturation_mean, texture_gray_std, texture_q90_q10 | Logistic Regression / Random Forest / Extra Trees | ~161 | ~40 |
| BreathRiskGOUT | 456 | 30 | Top 30 features from combined breath VOC (451) + clinical (5) pool; fold-specific selection via SelectKBest | Logistic Regression / Random Forest / Extra Trees | ~161 | ~40 |
| BreathGOUT-Plus | 484 | 30 | Top 30 features from combined breath VOC (451) + clinical (5) + tongue-image (28) pool; fold-specific selection via SelectKBest | Logistic Regression / Random Forest / Extra Trees | ~161 | ~40 |

Note:All models were trained and tested within a 5-fold stratified cross-validation framework (n = 201; ≈161 training / ≈40 test participants per fold; case–control ratio preserved in each fold). Feature selection (SelectKBest, k = 30) and preprocessing were performed exclusively within each training fold and applied unchanged to the corresponding test fold; reported metrics are based on pooled out-of-fold predictions. m/z, mass-to-charge ratio; VOC, volatile organic compound.

V.Appendix Supplementary Table 2

Supplementary Table 2.Comparison of 28 tongue features between gout patients and healthy control group

| **Feature** | **Healthy median** | **Gout median** | **r** | **P value** | **FDR q** |
| --- | --- | --- | --- | --- | --- |
| Tongue body whiteness index | 0.917 | 0.927 | 0.275 | <0.001 | <0.001 |
| Tongue body redness index | 0.168 | 0.149 | -0.266 | <0.001 | <0.001 |
| Tongue body saturation mean | 0.306 | 0.285 | -0.231 | 0.001 | 0.0043 |
| Tongue body texture q90 q10 | 0.387 | 0.429 | 0.201 | 0.004 | 0.03 |
| Tongue body texture gray std | 0.151 | 0.16 | 0.182 | 0.01 | 0.049 |
| Tongue body yellow coating index | 0.105 | 0.092 | -0.181 | 0.01 | 0.049 |
| Tongue body r std | 0.155 | 0.169 | 0.176 | 0.013 | 0.051 |
| Sublingual r mean | 0.599 | 0.567 | -0.173 | 0.014 | 0.051 |
| Tongue body r mean | 0.655 | 0.631 | -0.166 | 0.018 | 0.056 |
| Tongue body b std | 0.156 | 0.16 | 0.164 | 0.02 | 0.056 |
| Tongue body g std | 0.152 | 0.165 | 0.161 | 0.023 | 0.057 |
| Sublingual whiteness index | 0.911 | 0.919 | 0.147 | 0.037 | 0.08 |
| Sublingual darkness index | 0.521 | 0.546 | 0.147 | 0.037 | 0.08 |
| Sublingual redness index | 0.183 | 0.167 | -0.142 | 0.044 | 0.089 |
| Sublingual g mean | 0.426 | 0.41 | -0.117 | 0.096 | 0.18 |
| Sublingual yellow coating index | 0.105 | 0.103 | -0.113 | 0.108 | 0.189 |
| Sublingual b mean | 0.409 | 0.39 | -0.105 | 0.139 | 0.228 |
| Sublingual texture q90 q10 | 0.435 | 0.446 | 0.08 | 0.254 | 0.38 |
| Sublingual saturation mean | 0.348 | 0.341 | -0.08 | 0.258 | 0.38 |
| Sublingual r std | 0.166 | 0.169 | 0.072 | 0.307 | 0.429 |
| Tongue body darkness index | 0.461 | 0.47 | 0.069 | 0.33 | 0.44 |
| Sublingual texture gray std | 0.165 | 0.168 | 0.054 | 0.447 | 0.569 |
| Sublingual b std | 0.161 | 0.16 | 0.035 | 0.625 | 0.761 |
| Sublingual g std | 0.175 | 0.175 | 0.019 | 0.792 | 0.924 |
| Sublingual tongue mask ratio | 0.967 | 0.958 | -0.012 | 0.863 | 0.967 |
| Tongue body g mean | 0.494 | 0.489 | -0.003 | 0.966 | 0.978 |
| Tongue body tongue mask ratio | 0.969 | 0.971 | -0.003 | 0.969 | 0.978 |
| Tongue body b mean | 0.465 | 0.463 | 0.002 | 0.978 | 0.978 |

Note:Values are group medians. Group differences were assessed by the Mann-Whitney U test with Benjamini-Hochberg false discovery rate (FDR) correction across all 28 features. r, rank-biserial correlation (effect size). FDR q < 0.05. Features are ordered by raw P value. Tongue-body and sublingual features were extracted separately and aggregated per subject.
